# Supplementary material for: Association between Dietary Patterns and Chronic Obstructive Pulmonary Disease in Korean Adults: The Korean Genome and Epidemiology Study
Source: Nutrients. 2021 Dec 2;13(12):4348. doi: 10.3390/nu13124348 (PMC8707827; doi:10.3390/nu13124348)
Supplement: Supplementary file 1 [file nutrients-13-04348-s001.zip › nutrients-1470238-SI.pdf]

Supplementary Table S1. Baseline characteristics according to FEV1 by gender

Variables	Quartile of FEV1, %Pred. (Men = 2,599)					Quartile of FEV1, %Pred. (Women = 2,837)				
	Q1 (n=913)	Q2 (n=730)	Q3 (n=609)	Q4 (n=347)	*p*	Q1 (n=455)	Q2 (n=669)	Q3 (n=714)	Q4 (n=999)	*p*
Age, years	54.3±8.0	52.9±7.7	54.3±7.8	56.1±8.3	<0.001	53.6±8.1	53.6±7.7	54.5±8.2	57.0±8.7	<0.001
Smoking history, n (%)	646 (70.8)	466 (63.8)	353 (58.0)	191 (55.0)	<0.001	9 (2.0)	6 (0.9)	10 (1.4)	9 (0.9)	0.280
Height, cm	167.1±5.5	167.7±5.8	167.4±5.7	166.2±5.9	<0.001	155.6±5.3	155.1±5.1	154.5±5.3	153.0±5.7	<0.001
Weight, kg	68.5±9.1	68.8±9.1	68.5±9.0	65.9±8.7	<0.001	60.2±8.6	59.4±7.9	59.1±7.7	57.9±8.0	<0.001
Waist circumference, cm	86.1±7.5	84.9±7.0	84.8±7.4	82.9±7.2	<0.001	83.5±9.9	82.9±9.1	82.6±9.2	82.9±9.3	0.450
Body mass index, kg/m^2	24.6±2.8	24.5±2.6	24.4±2.7	23.9±2.7	0.001	24.9±3.4	24.8±3.1	24.8±3.0	24.7±3.1	0.830
<18.5, n (%)	15 (1.6)	8 (1.1)	6 (1.0)	6 (1.7)	0.020	7 (1.5)	3 (0.5)	4 (0.6)	12 (1.2)	0.330
18.5-25, n (%)	507 (55.5)	407 (55.8)	346 (56.8)	228 (65.7)		241 (53.0)	380 (56.8)	398 (55.7)	541 (54.2)	
≧25, n (%)	391 (42.8)	315 (43.2)	257 (42.2)	113 (32.6)		207 (45.5)	286 (42.8)	312 (43.7)	446 (44.6)	
Fat mass index, kg/m^2	5.5±1.7	5.2±1.6	5.1±1.6	4.9±1.6	<0.001	7.9±2.3	7.8±2.2	7.8±2.1	7.7±2.2	0.380
Muscle mass index, kg/m^2	18.1±1.5	18.2±1.4	18.3±1.4	18.0±1.4	0.001	16.0±1.4	16.0±1.2	16.0±1.2	16.0±1.2	0.820
Fat mass, kg	15.3±4.8	14.7±4.5	14.4±4.5	13.5±4.3	<0.001	19.1±5.3	18.8±5.2	18.5±4.9	18.0±5.0	0.001
Lean mass, kg	50.5±5.7	51.4±5.9	51.3±5.8	49.7±5.7	<0.001	38.9±4.4	38.5±3.9	38.3±3.9	37.7±4.2	<0.001
hsCRP, mg/L	2.1±4.8	1.3±2.1	1.4±3.8	1.3±2.5	<0.001	1.6±2.4	1.3±2.9	1.4±3.7	1.2±2.6	0.260
Total energy, kcal/day	1904.6±559.7	1925.1±491.9	1935.2±544.2	1908.2±555.1	0.700	1743.5±521.9	1680.1±494.8	1702.8±523.0	1656.5±496.8	0.020
% of energy from carbohydrate	71.3±6.6	71.2±6.0	70.9±6.3	71.9±6.1	0.100	72.8±7.2	73.1±6.3	73.2±6.3	74.1±6.2	<0.001
% of energy from protein	13.0±2.2	13.1±2.2	13.1±2.1	12.9±2.2	0.600	13.2±2.7	13.0±2.4	13.0±2.3	12.7±2.3	0.010
% of energy from fat	14.3±5.3	14.4±4.7	14.7±5.0	13.9±4.8	0.080	13.0±5.6	12.8±5.0	12.8±5.0	12.0±5.0	0.001
Protein, g/day	62.7±24.2	63.5±20.7	63.8±22.4	62.1±22.6	0.650	58.3±25.9	55.2±21.4	56.1±24.5	53.1±20.5	0.001
Fat, g/day	31.5±19.1	31.5±15.0	32.7±17.0	30.5±17.0	0.270	26.4±17.8	24.6±14.3	25.4±16.1	23.0±14.3	<0.001
Carbohydrate, g/day	336.6±93.0	340.8±84.1	340.7±92.8	340.7±93.9	0.740	313.8±85.4	305.3±86.9	308.6±85.4	305.0±86.1	0.280
Calcium, mg/day	418.3±232.7	421.1±217.6	432.8±230.0	443.3±233.1	0.270	445.4±258.3	440.5±263.5	448.3±258.0	422.2±242.9	0.140
Iron, mg/day	10.0±4.3	10.0±3.9	10.1±4.2	10.0±4.1	0.950	9.9±4.7	9.5±4.4	9.7±4.5	9.2±4.3	0.040
Vitamin A, RE/day	465.0±307.6	464.5±303.4	484.4±321.0	485.3±291.0	0.460	478.6±373.5	466.1±359.7	467.6±360.4	448.2±322.0	0.410
Sodium, mg/day	2818.0±1526.1	2780.2±1413.4	2851.3±1387.0	2852.2±1372.4	0.800	2597.9±1473.2	2467.4±1513.4	2521.0±1415.7	2540.4±1462.6	0.520
Vitamin B1, mg/day	1.1±0.5	1.1±0.4	1.1±0.4	1.1±0.4	0.690	1.0±0.4	0.9±0.4	1.0±0.4	0.9±0.4	0.002
Vitamin B2, mg/day	0.9±0.4	0.9±0.4	1.0±0.4	1.0±0.4	0.300	0.9±0.5	0.9±0.4	0.9±0.4	0.8±0.4	0.020
Vitamin C, mg/day	96.7±59.3	99.7±54.9	99.4±55.4	104.0±58.3	0.240	111.8±67.3	107.0±63.2	110.1±65.7	107.0±66.2	0.490
Zinc, mg/day	8.3±3.8	8.4±2.9	8.4±3.1	8.2±2.9	0.950	7.5±3.0	7.3±2.6	7.4±3.0	7.1±2.8	0.030
Vitamin B6, mg/day	1.6±0.6	1.7±0.6	1.6±0.6	1.7±0.6	0.930	1.6±0.7	1.5±0.6	1.6±0.7	1.5±0.6	0.050
Folate, ug/day	214.0±111.7	217.8±106.1	221.4±111.4	224.4±109.7	0.390	226.8±129.7	217.8±127.3	220.7±120.7	211.4±111.5	0.130
Fiber, g/day	5.9±2.7	5.9±2.5	6.0±2.5	6.1±2.5	0.480	6.0±2.8	5.8±2.7	5.9±2.7	5.8±2.7	0.490
Vitamin E, mg/day	8.2±4.0	8.3±3.7	8.5±4.0	8.4±4.0	0.480	8.2±4.3	7.9±4.1	8.0±4.3	7.6±4.1	0.090
Cholesterol, mg/day	162.8±120.9	162.1±106.6	165.5±111.4	158.0±118.8	0.810	149.7±121.9	140.8±112.5	144.5±120.6	130.7±106.8	0.010

Data are presented as mean±standard error or the number of cases (percentage). Abbreviations: FEV1, forced expiratory volume in one second; %Pred, % of predicted value; hsCRP, high sensitivity C-reactive protein

Supplementary Table S2. Comparison of daily food group intakes (g/day) between normal group and COPD group

Food groups	Model 1 (Crude model)						Model 2 (Energy-adjusted model*)					
	Men (n=2,599)			Women (n=2,837)			Men (n=2,599)			Women (n=2,837)		
	Normal (n=2,172)	COPD (n=427)	*p*	Normal (n=2,735)	COPD (n=102)	*p*	Normal (n=2,172)	COPD (n=427)	*p*	Normal (n=2,735)	COPD (n=102)	*p*
White rice, g/day	189.7±320.3	203.5±327.5	0.420	97.0±233.4	57.2±178.3	0.030	188.8±326.8	201.1±335.9	0.480	95.5±234.2	53.9±167.9	0.020
Whole grains, g/day	499.2±344.3	482.1±342.3	0.350	510.9±275.5	548.8±247.2	0.170	505.0±344.0	515.9±367.3	0.550	522.2±275.0	588.1±253.5	0.020
Noodle & Dumpling, g/day	70.1±71.6	70.0±72.0	0.980	44.7±59.7	32.0±55.7	0.030	70.9±69.9	72.5±66.0	0.680	44.7±55.4	38.2±57.1	0.250
Rice cakes, g/day	4.9±11.5	6.2±40.0	0.490	7.3±19.2	5.5±9.0	0.060	5.0±8.6	5.5±18.1	0.620	7.1±12.4	6.2±9.1	0.320
Cereals and Snacks, g/day	2.9±7.6	2.4±8.1	0.290	3.1±8.3	2.5±6.8	0.400	3.2±6.1	3.1±7.5	0.690	3.4±6.5	3.1±6.1	0.610
Bread, g/day	13.1±24.0	13.9±34.4	0.670	12.4±24.4	9.0±19.9	0.090	12.2±18.9	13.4±25.5	0.330	11.5±17.7	8.4±13.5	0.030
Pizza and hamburger, g/day	2.5±9.6	1.7±6.9	0.020	3.0±11.2	0.7±2.4	<0.001	3.2±8.1	2.5±6.2	0.050	3.6±9.2	1.7±2.8	<0.001
Potatoes and sweet potatoes, g/day	11.8±18.0	10.9±12.7	0.240	16.7±23.3	20.3±24.3	0.120	11.7±15.9	11.6±13.3	0.910	16.6±21.1	21.6±25.7	0.060
Starch jelly, g/day	2.0±6.1	1.5±3.1	0.004	2.2±6.2	1.4±2.8	0.005	2.6±5.5	2.1±2.8	0.020	2.7±4.8	2.1±2.7	0.040
Sweets, g/day	8.9±9.2	9.9±10.2	0.050	5.6±6.5	5.7±5.9	0.780	9.0±8.9	10.4±10.0	0.006	5.9±6.4	6.7±7.3	0.230
Nuts, g/day	0.9±2.2	0.8±1.9	0.060	0.9±3.0	0.6±1.4	0.020	1.5±2.0	1.3±1.6	0.180	1.5±2.6	1.2±1.2	0.020
Legumes, g/day	37.6±53.3	37.2±56.3	0.870	33.5±46.2	35.8±43.0	0.620	36.9±45.9	38.3±49.2	0.580	33.1±43.5	38.2±46.7	0.250
Vegetables, g/day	83.0±64.8	80.5±68.1	0.460	92.2±84.6	88.4±80.9	0.660	82.4±57.7	81.4±56.1	0.750	91.3±70.3	90.4±64.2	0.900
Kimchi, g/day	176.8±123.9	186.4±137.7	0.180	156.8±127.7	174.6±132.5	0.170	179.7±125.1	195.7±141.2	0.030	159.4±125.1	189.7±147.5	0.040
Mushrooms, g/day	6.8±8.7	5.3±8.8	0.001	7.3±10.7	6.1±11.0	0.280	6.7±7.8	5.5±7.2	0.001	7.3±9.4	6.2±9.7	0.230
Fruits, g/day	187.3±177.7	151.3±152.2	<0.001	235.7±205.6	184.4±144.9	0.001	180.7±150.9	154.3±132.8	<0.001	237.9±201.9	195.3±133.5	0.002
Meat and its products, g/day	56.1±49.4	51.8±71.4	0.230	33.2±40.7	39.6±75.8	0.400	55.2±41.5	51.7±39.6	0.100	31.7±28.8	35.8±33.8	0.230
Eggs, g/day	11.9±15.4	11.8±17.6	0.940	10.2±14.7	8.8±12.4	0.270	11.9±14.1	12.7±19.3	0.400	10.2±13.2	8.9±10.7	0.230
Fish and shellfish, g/day	36.5±32.0	31.4±33.8	0.004	32.8±36.8	29.3±34.9	0.350	35.6±28.7	32.8±32.5	0.100	31.8±29.0	29.2±27.6	0.370
Seaweeds, g/day	1.3±1.5	1.3±1.7	0.950	1.8±2.2	2.1±2.7	0.350	1.4±1.5	1.4±1.6	0.490	1.8±2.1	2.1±1.9	0.230
Dairy products, g/day	105.2±136.2	81.4±107.4	<0.001	123.8±140.8	127.9±154.9	0.770	102.6±122.6	88.5±131.4	0.040	126.5±148.1	139.4±169.6	0.450
Soup, g/day	3.9±4.0	4.4±4.8	0.050	3.9±4.2	4.4±4.6	0.190	3.9±3.9	4.6±4.8	0.010	3.9±4.4	4.7±4.8	0.100
Seasoning, g/day	0.1±0.7	0.1±0.7	0.840	0.2±0.9	0.2±1.1	0.840	1.0±0.6	1.0±0.6	0.240	1.0±0.9	1.1±1.0	0.430

Oils and fats, g/day	5.5±6.5	5.6±6.7	0.660	2.9±4.1	3.1±4.2	0.650	5.7±6.0	6.0±6.2	0.320	3.4±3.9	3.9±4.6	0.360
Coffee, g/day	5.0±5.0	5.3±5.2	0.320	3.2±3.4	2.9±3.0	0.500	5.2±4.8	5.6±4.9	0.120	3.5±3.3	3.5±3.3	0.960
Carbonated beverages, g/day	17.4±56.2	21.5±90.0	0.360	9.1±40.7	9.0±43.2	0.980	17.0±48.2	18.9±62.5	0.550	9.1±35.0	10.0±47.1	0.850
Other beverages, g/day	113.7±170.7	91.2±178.7	0.010	104.3±163.2	77.5±163.5	0.100	114.8±193.0	88.4±163.2	0.003	107.9±203.9	89.9±203.9	0.380

Data are presented as mean±standard error.

*Energy adjusted for Residual method

Abbreviations: COPD, chronic obstructive pulmonary disease
